# Evaluation of Targeted-Release Capsule Formulations for Protection of the Acid-Sensitive Enzyme Pancreatin Under Fasted and Fed Intestinal Conditions In Vitro

**DOI:** 10.3390/pharmaceutics18030285

**Published:** 2026-02-25

**Authors:** Elnaz Karimian Azari, Marlies Govaert, Cindy Duysburgh, Stanislaw Glab, Massimo Marzorati, Zainulabedin Saiyed

**Affiliations:** 1 Lonza Greenwood LLC, 535 N Emerald Road, Greenwood, SC 29646, USA; elnaz.karimianazari@lonza.com (E.K.A.); stan.glab@yahoo.com (S.G.); 2 ProDigest, Technologiepark 82, 9052 Zwijnaarde, Belgium; marlies.govaert@prodigest.eu (M.G.); cindy.duysburgh@prodigest.eu (C.D.); massimo.marzorati@prodigest.eu (M.M.); 3 Center for Microbial Ecology and Technology (CMET), Faculty of Bioscience Engineering, University of Ghent, Frieda Saeysstraat 1, 9052 Gent, Belgium

**Keywords:** DUOCAP^®^ capsule-in-capsule technology, drug delivery, digestive enzymes, targeted delivery, upper gastrointestinal tract simulation

## Abstract

**Objective:** This study assessed the ability of capsule formulations to improve the oral delivery and retain activity of an acid-sensitive enzyme during gastrointestinal transit. **Methods:** The dissolution characteristics of five capsule formulations—single DRcaps^®^ [DR], single Vcaps^®^ Plus [VCP], and three DUOCAP^®^ capsule-in-capsule combinations, DRcaps^®^ inside DRcaps^®^ (DR-in-DR), DRcaps^®^ inside Vcaps^®^ Plus (DR-in-VCP), and Vcaps^®^ Plus inside DRcaps^®^ (VCP-in-DR)—were evaluated in an in vitro simulation of a healthy human upper gastrointestinal tract under fasting and fed conditions using the Simulator of the Human Intestinal Microbial Ecosystem (SHIME)^®^ platform. Capsules contained caffeine as a marker of capsule dissolution, and pancreatin as an active ingredient for which activity was determined by the conversion of tributyrin. Readouts included visual capsule scoring, the analysis of caffeine release, and the quantification of tributyrin-to-butyrate conversion at the end of each gastrointestinal tract segment. **Results:** The single VCP capsules had a high level of caffeine release at the end of the stomach incubation with low butyrate recovery (16–21%), suggesting the rapid release and gastric degradation of the unprotected enzyme. The single DR, DR-in-VCP, and VCP-in-DR formulations showed caffeine release at the end of the duodenum and/or jejunum and had high butyrate recovery, ranging from 53% to 87%. The DR-in-DR formulation had the most delayed release, with incomplete caffeine release and low-to-moderate butyrate recovery (10–36%). **Conclusions:** Fast capsule dissolution led to the reduced enzymatic activity of the active ingredient, while delayed dissolution resulted in inadequate time for the enzymatic conversion of tributyrin to butyrate. These results highlight that capsule selection should align with the intended use and targeted nutrient delivery, with DUOCAP^®^ formulations being best suited for small intestinal (VCP-in-DR and DR-in-VCP) and colonic (DR-in-DR) delivery.

## 1. Introduction

The oral delivery of bioactives and dietary supplements is preferred over other methods considering their potential for targeted delivery, capability for the inclusion of solid formulations, and convenience [1]. However, oral delivery has some challenges, including potential damage to the product due to acidic pH conditions in the stomach or enzymatic degradation in the proximal small intestine [2,3]. Nutritional ingredients and bioactives including enzymes may become denatured or depurinated in low pH conditions, or may be degraded by gastric enzymes, resulting in decreased activity and effectiveness [4,5]. Thus, for many nutritional ingredients and dietary supplements, ensuring protected transit through the low pH environment of the stomach is necessary. Furthermore, optimizing the site of active ingredient(s’) release within the gastrointestinal tract is expected to improve the activity and overall effectiveness of these compounds.

Capsule technology has evolved to address these potential challenges with the oral delivery of dietary supplements and bioactives. Alternative polymers such as hydroxypropyl methylcellulose (HPMC), alginate, and pullulan, have begun to replace gelatin capsules, which has resulted in the possibility of site-specific bioactive delivery [6]. Other technological developments include advanced coating methods and the process of double dipping [6]. The DUOCAP^®^ system, where one capsule containing the bioactive compound is encapsuled by a second capsule (i.e., capsule-in-capsule technology), is another advancement [7,8,9,10,11]. This system improves the capsule disintegration time and enables targeted delivery by combining capsules with different dissolution characteristics. In this study, we investigated DRcaps^®^ (DR) and Vcaps^®^ Plus (VCP) within the DUOCAP^®^ system. DRcaps^®^ are composed of combined HPMC and gellan gum, and support delayed release in the small intestine [12,13,14,15]. Vcaps^®^ Plus are composed of HPMC and are manufactured using a thermal gelling process with a hot dip method, and have exhibit release in acidic environments [16]. Previous work has demonstrated that a combination of the DRcaps^®^ capsule in DRcaps^®^ capsule within the DUOCAP^®^ system improved the survivability of the probiotic strain *Lactobacillus acidophilus* in different parts of the gastrointestinal tract, supporting the delivery of viable and functional probiotics to the colon [7]. To enable the strategic use of the DUOCAP^®^ system for the optimal delivery of dietary supplements and nutritional ingredients, it is important to understand the dissolution profile of different capsule formulations during gastrointestinal transit, and under different conditions, such as fasted and fed states. These characteristics affect both the bioavailability and effectiveness of encapsulated bioactives. When individual properties of the bioactives are considered, capsule dissolution data provide critical guidance for selecting optimal capsule types and administration recommendations (e.g., whether to take with or without a meal).

Following their transit through the stomach, oral nutrients enter the small intestine where most food digestion and absorption take place. The supplementation of digestive enzymes for food-related applications, such as lipid digestion, should therefore focus on the small intestine. In this region, bile salts stabilize emulsified lipids before their digestion by pancreatic lipase [17], which is most active at a near-neutral pH [18]. Pancreatin, a concentrated enzyme mixture containing the pancreatic enzymes lipase, protease, and amylase [19], is active primarily in the duodenum and jejunum [20] and can convert tributyrin to butyrate [11]. These properties have been harnessed to allow for the quantification of enzymatic activity in specific intestinal regions during an in vitro simulation of the upper gastrointestinal tract, as such measurements are not feasible in vivo. Animal models offer limited utility due to considerable physiological differences in humans (e.g., pH, bile salt levels, retention time, and temperature). Thus, in vitro models that accurately replicate the physiological conditions of the human upper gastrointestinal tract and enable non-invasive sampling across all regions are valuable research tools. The Simulator of the Human Intestinal Microbial Ecosystem (SHIME)^®^ platform is a validated in vitro simulation of the physiological, chemical, and microbiological properties of the gastrointestinal tract [21]. This platform facilitates mechanistic studies in the stomach, small intestine, and colon.

This study aimed to assess the ability of five different capsule formulations (single capsules or a combination of capsules using the DUOCAP^®^ system), to improve oral delivery and retain the activity of an acid-sensitive enzyme when passing through the stomach and small intestine, in order to substantiate a cause-and-effect relationship between the kinetics of capsule disintegration and the release of an active following enzymatic degradation. Stomach and small intestinal conditions were simulated using an in vitro upper gastrointestinal tract model employing the SHIME^®^ platform. The effect of food was also assessed by testing each capsule formulation under both fasted and fed conditions for healthy adults.

## 2. Materials and Methods

### 2.1. Capsule Test Products

The capsule formulations utilized DR (composed of HPMC and gellan gum) and VCP (composed of HPMC manufactured with a thermal gelling process) capsules (Lonza Greenwood LLC., Greenwood, SC, USA). The DR-in-DR, DR-in-VCP, VCP-in-DR, single DR, and single VCP capsules each contained a powder mixture of 150 mg pancreatin (8 × USP, Sigma-Aldrich, Saint-Quentin-Fallavier, France) and 50 mg caffeine (Reagent Plus, Sigma-Aldrich, Saint-Quentin-Fallavier, France). The two powders were uniformly mixed and then encapsulated using a TORPAC ProFill100 Capsule Filling System (Fairfield, NJ, USA). After filling, each capsule was weighed and only the ones meeting the 200 mg target (+/−5%) were sealed and packaged. All capsules that contained the powder formulation were size #3 and in the case of the DR-in-DR, DR-in-VCP, and VCP-in-DR DUOCAP^®^ formulations, one capsule (#3) was manually over-encapsulated in a #00 capsule size. The capsule shell’s thickness was 100 µm (±10%), which is the same as for standard capsules of all sizes.

### 2.2. In Vitro Human Upper Gastrointestinal Tract Simulation Under Healthy Fed and Fasted Conditions

The upper gastrointestinal tract simulation was conducted according to Marzorati et al. [7], simulating healthy human conditions. Briefly, the stomach and small intestine digestion conditions were simulated sequentially in a double-jacketed reactor. For all the simulations, the reactor temperature was 37 °C and the contents were subjected to continuous magnetic stirring at 300 rpm [7]. Fasted (i.e., the consumption of the product before a meal) and fed (i.e., the consumption of the product with a meal) conditions were used for this study. All the experimental conditions were selected based on the InfoGest consensus method [22], though including dynamic pH profiles to better reproduce in vivo conditions. At the start of the stomach’s digestion, a single capsule formulation was added to each reactor using capsule sinkers designed for dissolution studies (ProSense, Oosterhout, The Netherlands). The capsules were positioned in the middle of the liquid, perpendicular to the stirring direction. This positioning resulted in the exposure of the capsules to shear stress, which mimicked the mechanical forces encountered during in vivo digestion. Additionally, 2.67 mL of tributyrin was added to each reactor at the start of the stomach’s digestion as the lipid source to measure pancreatin activity. Considering that lipases may be inhibited by excessive lipid concentrations, we used a conservative amount of tributyrin to mitigate the possibility of lipase inhibition. Stomach digestion was simulated with a 45 min incubation at pH 2.0 for the fasted condition [23] and a 2 h incubation with a sigmoidal decrease in pH from 5.5 to 2.0 for the fed condition [22]. For the fasted condition, 1000 U/mL pepsin and 0.02 mM phosphatidylcholine were added to a background medium containing only salts (NaCl, 50 mM; KCl, 7 mM) and mucins. For the fed condition, 4000 U/mL pepsin and 0.08 mM phosphatidylcholine were added to SHIME^®^ nutritional medium with protein and carbohydrate sources (PD01, ProDigest, Ghent, Belgium) and a salt level of 50 mM NaCl and 7 mM KCl. The samples were collected at the start and end of the stomach digestion (start: both conditions at 0 min; end: fasted condition at 45 min and fed condition at 120 min). Next, simulated pancreatic juice, containing pancreatic enzymes and bile salts (fasted condition: 3.1 TAME U/mL trypsin, 0.76 BTEE U/mL chymotrypsin and 3.33 mM bile salts; fed condition: 15.4 TAME U/mL trypsin, 3.8 BTEE U/mL chymotrypsin and 10 mM bile salts), was added to the reactor (reaching a final concentration of 15% *v*/*v*) to initiate the small intestinal incubation. To simulate the duodenal digestion, the pH was gradually increased from 2 to 6.5 and then maintained at 6.5 over a 27 min period. Next, the jejunal digestion was simulated by a stepwise pH increase to pH 7.5 over the course of 63 min. Finally, ileal digestion was simulated by maintaining the pH at 7.5 for 90 min. The capsules were incubated for a total of 225 min in the fasted condition (45 min stomach incubation plus 180 min total intestinal incubation) and 300 min in the fed condition (120 min stomach incubation plus 180 min total intestinal incubation). A negative control incubation, containing tributyrin but without capsules (and thus without caffeine and pancreatin) was included in the study design. All the test conditions (including the negative control) were performed in triplicate (n = 3) to account for variability.

### 2.3. Study Endpoints

#### 2.3.1. Visual Capsule Scoring

A visual inspection of the capsules was performed at distinct time points (i.e., stomach start, stomach end, duodenum end, jejunum end, and ileum end) to study their disintegration behavior during their passage through the different regions of the upper gastrointestinal tract. Scores were given using the following criteria: a score of one was given if the capsule was intact, a score of two if the capsule was damaged but almost all of the powder contents (>75%) was still inside, a score of three if the capsule was damaged with 50% of the powdered contents still inside, a score of four if the capsule was damaged with 25% of the powdered contents still inside, a score of five if the shell was still visible but all of the powdered contents had been released, and a score of six if the capsule was completely destroyed and the sinker was empty. For the DUOCAP^®^ formulations, double capsule scoring was applied, where both the inner and outer capsule received a separate capsule score on each time point.

#### 2.3.2. Analysis of Caffeine Release

Samples collected at the end of the stomach, duodenum, jejunum, and ileum incubations were evaluated for caffeine concentration using a method previously described in Marzorati et al. [7]. Briefly, HPLC-UV/Vis (Hitachi Chromaster HPLC-DAD, Hitachi High-Tech Corporation, Tokyo, Japan) was employed to measure the concentration of caffeine using an isocratic separation method (25% methanol:75% water [*v*/*v*]) on a Kinetex^®^ C18 LC column (5 μm, 100 Å, LC Column 100 × 4.6 mm, and solid support of Core–shell Silica) (Phenomenex, Woerden, The Netherlands) at a controlled column temperature of 30 °C. The injection volume was 10 μL and the per-sample run time was a total of 7 min. Caffeine was quantified using external standards (Sigma-Aldrich, Merck KGaA, Darmstadt, Germany). The samples were centrifuged for 7 min at 12,000 rpm prior to injection. Finally, the supernatant was filtered (0.2 μm) and analyzed. The limit of quantification (LOQ) for caffeine was 0.02 µg/mL; values below the LOQ were replaced with zero in the analysis.

#### 2.3.3. Tributyrin and Butyrate Analysis

The samples were collected and flash frozen (liquid nitrogen) at the end of the stomach, duodenum, jejunum, and ileum incubations for the evaluation of the tributyrin and butyrate concentrations as a measure of pancreatin enzymatic activity. Immediately prior to analysis, the frozen samples were thawed and diluted in water as necessary. The tributyrin and butyrate concentrations were determined as previously described [11]. Briefly, butyrate and tributyrin were extracted using acetonitrile, following the addition of 2-methyl hexanoic acid as an internal standard. The quantification was performed using a GC-2030 gas chromatograph (Shimadzu, ‘s-Hertogenbosch, The Netherlands), equipped with a capillary SH-PolarD column (dimensions: 30 mm × 0.32 mm × 1.00 µm; Shimadzu), a flame ionization detector and a split injector. The injection volume was 1 µL and the temperature profile was set from 110 to 160 °C with a temperature increase of 6 °C/min. The carrier gas was nitrogen, and the temperature of the injector and detector was set at 200 °C. The LOQ for tributyrin and butyrate was 0.1 mM and 0.25 mM, respectively; values below the LOQ were replaced by zero in the analysis.

### 2.4. Statistical Analysis

The percentages of caffeine and butyrate recovery are reported using descriptive statistics (mean ± standard deviation [SD]). The percentage of caffeine recovery was calculated by dividing the caffeine concentration measured at each time point by the concentration of caffeine included in each capsule (i.e., 50 mg). The percentage of butyrate recovery was calculated by dividing the butyrate concentration measured at each time point by the theoretical maximum concentration of butyrate that can be released from tributyrin at the end of the simulation (i.e., 18.2 mmol/reactor). Statistically significant differences in the released tributyrin and butyrate absolute concentrations between capsule formulations at the end of small intestine transit were determined using two-tailed homoscedastic Student’s *t*-tests. A *p*-value < 0.05 was considered significant with a confidence interval (CI) of 95%. The analyses were performed using GraphPad Prism software, version 10.6.0 (GraphPad Software, San Diego, CA, USA). Generative artificial intelligence was not used in this paper.

## 3. Results

### 3.1. Capsule Disintegration Behavior

Visual capsule scores during their passage through the upper gastrointestinal tract are shown in Table 1. Capsule scores ranged from one (capsule intact) to six (capsule destroyed; capsule sinker empty). Both the outer and inner DR-in-DR DUOCAP^®^ capsules remained intact through the stomach and duodenum incubations, and showed mild (scores of 1–2) signs of damage through the jejunum and ileum incubations. For the DR-in-VCP DUOCAP^®^ capsule, the outer capsule (VCP) was completely destroyed (score six) at the end of the stomach incubation, while the inner capsule (DR) began to show signs of damage at the end of the duodenum incubation; the damage progressed through the end of the jejunum and ileum incubations, with a final ileum score of four or three depending on the condition (i.e., fasted or fed). For the VCP-in-DR DUOCAP^®^ capsule, both the inner (VCP) and outer (DR) capsules remained intact at the end of the stomach incubation; the outer capsule began to show damage at the end of the duodenum incubation, which continued to progress through the end of the jejunum incubation (score of six or five depending on the condition) with considerable disintegration also obtained for the inner VCP capsule by the end of the ileum incubation (score of five or four depending on the condition). The single DR capsule was intact at the end of the stomach incubation and began to show progressive damage through the remaining incubations, while the single VCP capsule was destroyed at the end of the stomach incubation. In all cases, the DR capsule was more robust compared to the VCP capsule.

When comparing the capsule scores for both nutritional states, capsule damage tended to occur more slowly in the fed versus fasted condition, except in the case of the VCP capsule in the DR-in-VCP DUOCAP^®^ or single VCP capsule formulations, where the VCP capsule was completely destroyed at the end of the stomach incubation for both experimental conditions.

### 3.2. Caffeine Release

**Figure 1:**
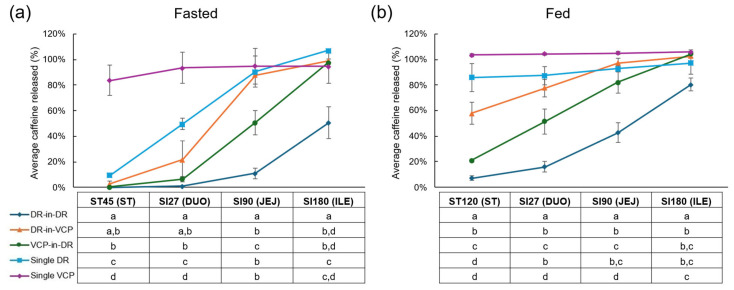
Effect of capsule formulation on caffeine release during passage through different regions of the gastrointestinal tract under fasted (**a**) and fed (**b**) conditions. Dots represent average caffeine recovery at different time points (%, *n* = 3). Error bars represent standard deviation. ST45 represents the end of the ST incubation (45 min) in the fasted condition and ST120 represents the end of the ST incubation (120 min) in the fed condition. For the SI incubations, SI27 represents the end of the DUO incubation (27 min), SI90 represent the end of the JEJ incubation (90 min), and SI120 represents the ILE incubation (120 min). Statistically significant differences (*p* < 0.05) between the capsule formulations at each time point are indicated by means of different letters. Average values below LOQ were not included in the statistical analysis. Student’s *t*-test was used to determine significant differences. DR = DRcaps^®^ capsule; DUO = duodenum; ILE = ileum; JEJ = jejunum; SI = small intestine; ST = stomach; and VCP = Vcaps^®^ Plus capsule.

Caffeine release was used as an active marker of capsule dissolution during the simulated upper gastrointestinal transit. The caffeine release over time under fasted and fed conditions is shown in Figure 1a and Figure 1b, respectively. For all capsule formulations, except the DR-in-DR DUOCAP^®^, the caffeine was fully released by the end of the ileum incubation, with the release tending to be more gradual under fasted versus fed conditions. Caffeine release was most gradual for the DR-in-DR DUOCAP^®^ capsule formulation, where release was 0% and 1% at the end of the stomach and duodenum incubations, increasing to 11% at the end of the jejunum incubation, and further to 51% at the end of the ileum incubation under fasted conditions. Under fed conditions, small amounts were released at the end of the stomach (7%) and duodenum (16%) incubations, with further increases at the end of the jejunum (43%) and ileum (80%) phases. Under fasted conditions, caffeine release for the DR-in-VCP DUOCAP^®^ and VCP-in-DR DUOCAP^®^ was absent or negligible at the end of the stomach incubation, and was a respective 22% and 6% at the end of the duodenum incubation, 88% and 51% at the end of the jejunum, and 99% and 98% at the end of the ileum. The release was faster under fed conditions, with notable release starting at the end of the stomach incubation, especially for the DR-in-VCP DUOCAP^®^ formulation (stomach: 58% and 21%; duodenum: 78% and 51%; jejunum: 97% and 82%; and ileum: full release). While the caffeine release for the single DR capsule formulation was a respective 10%, 50%, and 90% at the end of the stomach, duodenum, and jejunum incubations, obtaining full release by the end of the ileum under fasted conditions, near complete release was already observed at the end of the stomach incubation under fed conditions (86%, 87%, 93%, and 97%, respectively). The single VCP capsule released caffeine most rapidly, with 84%, 94%, 95%, and 95% of the caffeine released at the end of the stomach, duodenum, jejunum, and ileum incubations, respectively, under fasted conditions, and all caffeine was released by the end of the stomach incubation under fed conditions.

### 3.3. Stability of Enzymatic Activity

**Figure 2:**
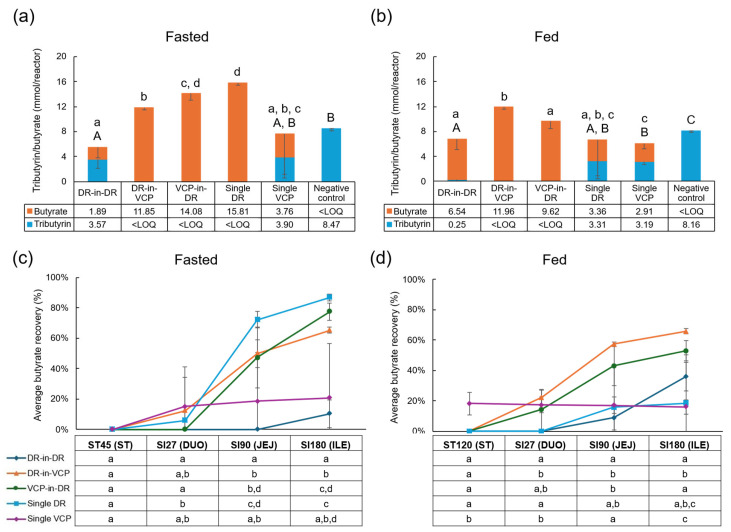
Effect of capsule formulation on tributyrin/butyrate release. Absolute values (mmol/reactor, *n* = 3) of tributyrin/butyrate release at the end of the small intestine under fasted (**a**) and fed (**b**) conditions, and average percentage of butyrate released during passage through different regions of the upper gastrointestinal tract under fasted (**c**) and fed (**d**) conditions. In panels (**a**,**b**), statistically significant differences (*p* < 0.05) between the different test conditions are indicated by means of different letters (small letters: butyrate content; capital letters: tributyrin content). Average values below LOQ were not included in the statistical analysis. Student’s *t*-test was used to determine significant differences between different test conditions. In panels (**c**,**d**), dots represent average butyrate recovery at different time points (%, *n* = 3). Error bars represent standard deviation. ST45 represents the end of the ST incubation (45 min) in the fasted condition and ST120 represents the end of the ST incubation (120 min) in the fed condition. For the SI incubations, SI27 represents the end of the DUO incubation (27 min), SI90 represents the end of the JEJ incubation (90 min), and SI120 represents the ILE incubation (120 min). Statistically significant differences (*p* < 0.05) between the capsule formulations at each time point are indicated by means of different letters. Average values below LOQ were not included in the statistical analysis. Student’s *t*-test was used to determine significant differences. DR = DRcaps^®^ capsule; LOQ = limit of quantification; DUO = duodenum; ILE = ileum; JEJ = jejunum; SI = small intestine; ST = stomach; and VCP = Vcaps^®^ Plus capsule.

The conversion of tributyrin to butyrate was used to determine pancreatin activity. Tributyrin was only detected for the negative control, confirming the absence of lipase activity under both experimental conditions. Under fasted conditions, only butyrate was detected at the end of the ileum incubation with the DR-in-VCP DUOCAP^®^, VCP-in-DR DUOCAP^®^, and single DR capsule formulations, indicating good pancreatin stability (Figure 2a). Both tributyrin and butyrate were detected with DR-in-DR DUOCAP^®^ and single VCP, indicating that pancreatin was less active in these capsule formulations. Under these fasted conditions, butyrate levels were significantly higher with the single DR capsule and VCP-in-DR DUOCAP^®^ compared with the other capsule formulations. Under fed conditions, butyrate alone was detected with the DR-in-VCP DUOCAP^®^ and VCP-in-DR DUOCAP^®^ capsule formulations, and butyrate with a small amount of tributyrin was detected with the DR-in-DR DUOCAP^®^ formulation, indicating good pancreatin activity with these formulations (Figure 2b). For both the single DR and single VCP capsules, similar amounts of tributyrin and butyrate were detected, indicating some loss of pancreatin activity. The DR-in-VCP DUOCAP^®^ resulted in the highest butyrate concentration, which was significantly higher than the DR-in-DR DUOCAP^®^, VCP-in-DR DUOCAP^®^, and single VCP capsule formulations. Although the average butyrate concentration was also numerically higher with the DR-in-VCP DUOCAP^®^ than the single DR, high variability in the concentration detected with the single DR negated a statistically significant difference.

The percent of butyrate recovery is shown in Figure 2c,d. Under fasted conditions, the single DR, VCP-in-DR DUOCAP^®^, and DR-in-VCP DUOCAP^®^ formulations resulted in higher butyrate recoveries (87%, 77%, and 65%, respectively) compared with the single VCP (21%), DR-in-DR DUOCAP^®^ (10%), and negative control (below LOQ), indicating a higher enzyme activity in the former versus latter capsule formulations (Figure 2c). Under fed conditions, the DR-in-VCP DUOCAP^®^ showed the highest average butyrate recovery (66%), followed by the VCP-in-DR DUOCAP^®^ (53%), DR-in-DR DUOCAP^®^ (36%), single DR (18%), single VCP (16%), and negative control (below LOQ) (Figure 2d). The butyrate recovery was higher overall under fasted versus fed conditions, with the exception of the DR-in-DR DUOCAP^®^ formulation.

## 4. Discussion

This study evaluated the ability of five capsule formulations to retain the enzymatic activity of an acid-sensitive enzyme when passing through an in vitro upper gastrointestinal tract simulation model under both fasted and fed conditions, focusing on the combined effect of protection of the capsule’s content and the release kinetics of the active compound. A visual capsule score and the caffeine release were used as quantitative markers to assess capsule disintegration. Overall, the study demonstrated clear differences in the timing of capsule dissolution and caffeine release, and varying levels of enzymatic activity over the course of the simulation, which were dependent on both the capsule combinations and nutritional state. The capsule combinations that released most of their caffeine contents by the end of the stomach incubation showed reduced enzymatic activity (single VCP; single DR, only fed). In contrast, formulations that retained caffeine through the end of the small intestine simulation showed lower enzymatic activity (DR-in-DR DUOCAP^®^), which suggests their potential suitability for colonic delivery. Finally, the other capsule combinations released most of the caffeine just before or during the jejunal incubation, which was associated with high enzymatic activity (single DR, only fasted; VCP-in-DR DUOCAP^®^; DR-in-VCP DUOCAP^®^). Collectively, our findings demonstrate that the tested technologies offer distinct release profiles and protection characteristics, which allow for targeted enzyme delivery.

A more detailed analysis of caffeine release kinetics confirmed this classification. Caffeine release data indicated that under both nutritional states, complete content release was observed for all formulations by the end of the simulation, except for the DR-in-DR DUOCAP^®^. A closer examination of the caffeine release profiles and corresponding butyrate recoveries revealed that the DR-in-DR DUOCAP^®^ formulation exhibited a delayed and incomplete release, resulting in relatively low butyrate recoveries. The single VCP and single DR (only fed) formulations showed content release during upper gastrointestinal transit, likely contributing to partial enzyme inactivation and low butyrate recoveries. These results are in line with a previous SHIME^®^ study that reported very low caffeine release in the stomach incubation with the DR-in-DR DUOCAP^®^, and fast release for single VCP capsules [7], and are further aligned with an in vivo study in fasting individuals that demonstrates capsule disintegration in the stomach for VCP capsules and the ileum for DR-in-DR DUOCAP^®^ [24]. VCP capsules are formulated entirely of HPCM, which is sensitive to pH, and are designed for immediate quick release, accounting for the rapid disintegration of the VCP capsules in the stomach [24]. The addition of gellan gum in the DR capsules protects HPMC from breaking down in the low pH environment of the stomach, allowing the intact capsules to transit to the intestines [24].

However, for the effective delivery of acid-sensitive enzymes, a precise release time profile is equally important as the protection of the capsule content during early gastrointestinal transit. Venkatesh et al. have shown that the optimal target site for pancreatin activity is towards the end of the jejunum [25], which was observed for the single DR (only fasted), VCP-in-DR DUOCAP^®^, and DR-in-VCP DUOCAP^®^ capsules during the present study. This allowed sufficient time for enzymatic conversion under the optimal pH, resulting in higher butyrate recoveries. The previous in vitro study also reported optimal content release timing for the single DR and VCP-in-DR DUOCAP^®^ capsule formulations under fasted conditions (DR-in-VCP DUOCAP^®^ was not tested), though showed a faster release early in the small intestinal incubation for the VCP-in-DR DUOCAP^®^ under fed conditions [7]. The present study’s findings are also generally consistent with an in vivo study reporting that for most participants (5/6), DR capsule disintegration occurred in the jejunum, and for the VPC-in-DR DUOCAP^®^ disintegration occurred in the jejunum or ileum for 3/6 and 2/6 participants, respectively [8].

The detailed quantification of butyrate further established the important relationship between the kinetics of capsule disintegration and the timely release of acid-sensitive enzymes, such as pancreatin. The maximum butyrate recoveries observed during upper gastrointestinal transit in the current study reached from 66% to 87%, which is in line with in vivo findings [26]. Butyrate data showed that under fasted conditions, the single DR, VCP-in-DR DUOCAP^®^, and DR-in-VCP DUOCAP^®^ capsules yielded the highest butyrate recoveries (87%, 77%, and 65%, respectively), likely due to complete enzyme release near the jejunal phase. In contrast, the single VCP and DR-in-DR DUOCAP^®^ formulations showed significantly lower recoveries due to fast or delayed release, respectively. Under fed conditions, the DR-in-VCP DUOCAP^®^ capsule formulations achieved the highest enzyme activity (butyrate recovery of 66%), followed by the VCP-in-DR DUOCAP^®^ (53%) and DR-in-DR DUOCAP^®^ (36%). Despite early gastric release, the DR-in-VCP DUOCAP^®^ benefited from full release before the end of the jejunum incubation, allowing sufficient enzymatic activity. The single DR and single VCP capsules again showed low recoveries due to early release during gastric transit and possible partial enzyme inactivation. These findings expand upon the results of the previous SHIME^®^ study, which showed the low recovery of the acid-sensitive probiotic *L. acidophilus* in the small intestine with the single VCP capsule and high recovery with the DR-in-DR DUOCAP^®^ formulation under both nutritional states [7]. Together, these studies highlight the DUOCAP™ system’s effectiveness in targeted delivery for a range of actives.

The different capsule formulations offered varying levels of protection during gastrointestinal transit, suggesting their potential for targeted content release. Other technologies, such as capsule shell coating, may also produce targeting effects. For example, the capsule used in the drug Nefecon^®^, which utilizes a capsule shell with a unique coating designed for delayed release, demonstrated consistent targeting of the contents to the distal ileum [27]. Capsule formulation also appears to have some effect on whether capsule disintegration is affected by nutritional state, with the DUOCAP^®^ formulations and the single VCP capsule demonstrating a consistent release profile in both nutritional states, in contrast to the differences observed with the single DR capsule. A consistent release profile between the fasted and fed states may be considered a desirable attribute and has also been observed with other capsule technologies, including the Enprotect^®^ bilayer capsule, which is made with HMPC and HPMC acetate succinate using a double-dipping process [28,29].

When considering the utilization of specific capsule formulations for targeted delivery, the findings for the DR-in-DR DUOCAP^®^ suggest that it may be useful for colonic targeting. This is supported by a study that evaluated the oral delivery of a fecal microbiome transplant (FMT) in patients with recurrent *Clostridium difficile* infection, using DR-in-DR DUOCAP^®^ to deliver the microbial contents of the FMT [30]. While capsule disintegration and release characteristics were not specifically evaluated, the study reported a 90% overall response rate to FMT, strongly suggesting successful colonic targeting. However, a study in human volunteers found that the DR-in-DR DUOCAP^®^ did not reach the colon, but rather the inner capsule reliably opened in the ileum [8].

This study was limited in that the study results cannot be directly extrapolated to the in vivo situation, as the results are based on an in vitro simulation. Additionally, no complementary methodologies, such as Fourier Transform Infrared Spectroscopy (FTIR), microscopy and/or photographic evidence, were included in the study design, which would be required to further substantiate claims regarding capsule disintegration. However, the inclusion of analytical markers in the current study still made it possible to assess the performance of the different capsule formulations. Finally, while the dose of tributyrin used in this study may be considered a low concentration, thereby optimizing enzyme-to-substrate exposure, we note that lipases may be inhibited by excessive lipid concentrations. To mitigate the possibility of lipase inhibition in this study, we used a conservative tributyrin concentration.

## 5. Conclusions

We conclude that the timing of capsule disintegration is a critical factor of butyrate recovery. Early disintegration during the stomach incubation exposes pancreatin to the acidic stomach environment, leading to partial inactivation. Conversely, delayed disintegration limits the time available for tributyrin into butyrate hydrolysis, reducing butyrate formation before completion of the upper gastrointestinal tract simulation. For the effective delivery of acid-sensitive digestive enzymes, depending on the suggested use and target site for enzyme delivery, using either the single DR, the DR-in-VCP DUOCAP^®^, or the VCP-in-DR DUOCAP^®^ capsule formulations is recommended due to their favorable release profile. However, the DR-in-DR DUOCAP^®^ capsule formulation is preferable for delayed release toward the colon. The results of the current study highlight that selecting the appropriate capsule formulations enables targeted delivery and tailored application.

## Figures and Tables

**Table 1:** Visual capsule scores during passage through different regions of the gastrointestinal tract.

	ST End (Outer/Inner)	DUO End (Outer/Inner)	JEJ End (Outer/Inner)	ILE End (Outer/Inner)
DR-in-DR				
Fasted	1/1	1/1	2 */1	2 */2
Fed	1/1	1/1	2 */2	2 */2
DR-in-VCP				
Fasted	6/1	6/1	6/4	6/4
Fed	6/1	6/2	6/2	6/3
VCP-in-DR				
Fasted	1/1	4/1	6/2	6/5
Fed	1/1	3/2	5/3	5/4
Single DR				
Fasted	1	2	4	4
Fed	1	3	4	4
Single VCP				
Fasted	6	6	6	6
Fed	6	6	6	6

DR = DRcaps^®^ capsule; DUO = duodenum; ILE = ileum; JEJ = jejunum; ST = stomach; and VCP = Vcaps^®^ Plus capsule. * capsule shell is visually not damaged, though swollen. Capsule scoring criteria: 1: Capsule intact. 2: Capsule damaged, with almost all product (>75%) still inside. 3: Capsule damaged, with 50% of the product still inside. 4: Capsule damaged, with 25% of product still inside. 5: Capsule shell visible, with all product released. 6: Capsule destroyed, and capsule sinker empty.

## Data Availability

The data supporting the findings of this study are available from the corresponding author on reasonable request.

## Abbreviations

The following abbreviations are used in this manuscript:

CI	Confidence interval
DR	DRcaps^®^
DUO	Duodenum
HPMC	Hydroxyl propyl methylcellulose
ILE	Ileum
JEJ	Jejunum
LOQ	Limit of quantification
SD	Standard deviation
SHIME^®^	Simulator of the Human Intestinal Microbial Ecosystem^®^
SI	Small intestine
ST	Stomach
VCP	Vcaps^®^ Plus
